# Death of a Medical Practitioner by Chloroform

**Published:** 1860-06

**Authors:** 


### Death of a Medical Practitioner by Chloroform.

We regret to have to announce another fatal accident during the administration of chloroform for the purpose of producing anæsthesia in a surgical operation. The unhappy patient was Dr. Renwick, of Alloa, a member of our own profession, and but twenty-seven years of age. His disease was ingrowing of the great toe-nail, which it was proposed to remedy by evulsion.—Dr. Renwick had previously inhaled chloroform without any bad result; hence, perhaps a false sense of security. In this unhappy case, also, the chloroform was administered in that dangerous manner to which we have heretofore adverted on more than one occasion. It was not given in measured dose by an inhaler, with a graduated admixture of atmospheric air. The circumstances are thus related:—

A little of the chloroform was poured upon a towel, and he held it to his mouth with his own hands. After awhile, as it did not seem to be taking any effect, he asked for some more which Dr. Duncanson at first declined to give; but after awhile, finding that no effect was being produced, some more was applied. Observing that he was endeavoring to hasten its effect by strained inspirations, he was asked to breathe naturally, which he did. As it still, however, seemed to be having no effect, another small quantity, at his own request, was applied to the towel, which after a time, produced insensibility; and his pulse having been found full and regular, the operation, which did not occupy more than a minute or two, was successfully performed.—He still remained under the influence of the anæsthetic, but his breathing was regular, and all was considered right. Some cold water was then thrown on his face to arouse him; but this not having the desired effect, other measures were resorted to, but with a like unfortunate result; and when, after a few minutes, his breathing became less frequent and more labored, and the appearance of his countenance began to change, and his pulse had become nearly imperceptible, serious alarm was felt. Artificial respiration by the modern method was resorted to, and in this manner breathing was kept up for nearly half an hour; but melancholy to relate, his spirit had passed away.—*Lancet.*

